# Prognostic stratification of sepsis through DNA damage response based RiskScore system: insights from single-cell RNA-sequencing and transcriptomic profiling

**DOI:** 10.3389/fimmu.2024.1345321

**Published:** 2024-02-09

**Authors:** Qingjiang Lin, Rongyao Zeng, Jinfeng Yang, Zebo Xu, Shaoxiong Jin, Guan Wei

**Affiliations:** Department of Emergency Surgery, The Second Affiliated Hospital of Fujian Medical University, Quanzhou, Fujian, China

**Keywords:** single-cell RNA-sequencing, DNA damage response, sepsis, prognostic model, immune response

## Abstract

**Background:**

A novel risk scoring system, predicated on DNA damage response (DDR), was developed to enhance prognostic predictions and potentially inform the creation of more effective therapeutic protocols for sepsis.

**Methods:**

To thoroughly delineate the expression profiles of DDR markers within the context of sepsis, an analytical approach utilizing single-cell RNA-sequencing (scRNA-seq) was implemented. Our study utilized single-cell analysis techniques alongside weighted gene co-expression network analysis (WGCNA) to pinpoint the genes that exhibit the most substantial associations with DNA damage response (DDR). Through Cox proportional hazards LASSO regression, we distinguished DDR-associated genes and established a risk model, enabling the stratification of patients into high- and low-risk groups. Subsequently, we carried out an analysis to determine our model’s predictive accuracy regarding patient survival. Moreover, we examined the distinct biological characteristics, various signal transduction routes, and immune system responses in sepsis patients, considering different risk categories and outcomes related to survival. Lastly, we conducted experimental validation of the identified genes through *in vivo* and *in vitro* assays, employing RT-PCR, ELISA, and flow cytometry.

**Results:**

Both single-cell RNA sequencing (scRNA-seq) and bulk transcriptomic analyses have demonstrated a strong correlation between DNA damage response (DDR) levels and sepsis prognosis. Specific cell subtypes, including monocytes, megakaryocytes, CD4+ T cells, and neutrophils, have shown elevated DDR activity. Cells with increased DDR scores exhibited more robust and numerous interactions with other cell populations. The weighted gene co-expression network analysis (WGCNA) and single-cell analyses revealed 71 DDR-associated genes. We developed a four-gene risk scoring system using ARL4C, CD247, RPL7, and RPL31, identified through univariate COX, LASSO COX regression, and log-rank (Mantel-Cox) tests. Nomograms, calibration plots, and decision curve analyses (DCA) regarding these specific genes have provided significant clinical benefits for individuals diagnosed with sepsis. The study suggested that individuals categorized as lower-risk demonstrated enhanced infiltration of immune cells, upregulated expression of immune regulators, and a more prolific presence of immune-associated functionalities and pathways. RT-qPCR analyses on a sepsis rat model revealed differential gene expression predominantly in the four targeted genes. Furthermore, ARL4C knockdown in sepsis model *in vivo* and vitro caused increased inflammatory response and a worse prognosis.

**Conclusion:**

The delineated DDR expression landscape offers insights into sepsis pathogenesis, whilst our riskScore model, based on a robust four-gene signature, could underpin personalized sepsis treatment strategies.

## Introduction

Sepsis, identified as a critical condition characterized by the body’s severe and imbalanced response to infection leading to widespread organ failure, stands as a principal cause of death and surging expenses in contemporary intensive care settings (1). Contemporary academic publications have indicated that the year 2017 witnessed approximately 48.9 million occurrences of sepsis and roughly 11 million deaths attributable to this condition worldwide. This represents nearly one-fifth of total global fatalities (2, 3). Over the past twenty years, strategies including controlling infections, administering fluid replacement therapy, and delivering support for multiple failing organs, have collectively contributed to the decline in death rates due to sepsis (4). Biomarkers have been evaluated for various functions in sepsis patients, including identifying infections, predicting prognosis, and informing treatment plans (5). Notwithstanding the limited exploration of patient variability in sepsis, definitive evidence showcasing the efficacy of tailored interventions aimed at distinct elements of the body’s reaction to sepsis in enhancing patient recovery remains elusive. Thus, to facilitate personalized treatment strategies for sepsis patients, a comprehensive understanding of the heterogeneity of sepsis and precise identification of each patient’s molecular features are essential.

The DNA Damage Response (DDR) functions as an inherent alarm system, perpetually safeguarding genome integrity and assuring the accurate transfer of genetic information to descendant cells. It has been noted from various studies that numerous pathophysiological conditions distinguished by elevated oxidative stress also demonstrate an amplified DDR regulation (6–8). Sepsis serves as a prime example of a condition characterized by heightened oxidative stress due to its inflammatory nature. Recent findings suggest that elements involved in DNA damage response (DDR), like ATM, p53, and p21, may act as catalysts for subsequent epigenetic alterations (9). Compared to healthy individuals, sepsis patients showed heightened oxidative DNA damage levels. A malfunctioning DDR could potentially precipitate excessive apoptosis and necrosis (10). Increased levels of plasma cell-free DNA (cfDNA) have been documented in several ailments where cell death and damage to tissue/organs catalyze pathogenesis, including sepsis (11). Moreover, a DNA damage response facilitated by TOP2A might contribute to the progression of ARDS precipitated by sepsis (12). Crucially, the orchestration of DNA damage response (DDR) may prove to be a compelling strategic point for intervention in treating sepsis. Hence, it is critically significant to comprehensively detail the regulatory elements associated with DDR and to unravel the fundamental molecular processes implicated in the development of sepsis.

In this study, we performed an in-depth examination of DDR-associated variant expression profiles in individuals with sepsis by applying a single-cell methodology. The study focused on the DDR framework within a range of cellular populations at distinct phases and prognostic points of sepsis, revealing the cellular interaction patterns within sepsis specimens exhibiting varying DDR intensities. Additionally, a Bulk-RNA-Seq-driven WGCNA was employed to pinpoint gene clusters linked to DDR, and characteristic DDR genes were pinpointed by overlapping single-cell with WGCNA data. Following this, we developed a risk assessment system and a predictive nomogram tailored to scrutinize biological characteristics, implicated pathways, prognostic survivals, and immune system signatures among sepsis sufferers stratified by risk. The expression levels and biological roles of these signature genes underwent validation through *in vivo* studies. Our research emphasizes the critical association between DDR expression profiles and the complexity of sepsis, offering distinctive perspectives on personalized prognostic categorization and therapeutic strategies for sepsis patients.

## Methods

### Raw data collection

Single-cell data was acquired from the peripheral blood of two healthy subjects and five hospitalized patients diagnosed with sepsis caused by gram-negative bacteria. This information is accessible on the GEO website under the accession number GSE167363. Bulk sepsis transcriptome data were also obtained (GSE65682, GSE95233, GSE63042, GSE95233, GSE106878, E-MTAB-5273, and E-MTAB-5274) from the GEO and ArrayExpress databases. Raw data underwent log2-transformation and normalization using the Robust Multiple Array Average (RMA) function with the “affy” R package.

### Single-cell RNA-seq data processing and cell annotation

The accuracy of the scRNA-seq data was verified using “Seurat” R tools. To maintain data integrity, we excluded genes that appeared in less than five individual cells, as well as cells that contained a gene count ranging from 200 to 3000 and those with mitochondrial gene content exceeding 15%. Consequently, a subset of 55,268 cells was identified and chosen for subsequent analytical procedures. After data normalization and scaling procedures on the remaining cell population with the “NormalizeData” and “ScaleData” techniques, we pinpointed the 3,000 genes exhibiting high variability by invoking the “FindVariableFeatures” function. To mitigate batch effects that may skew the subsequent analytical interpretations—owing to the diverse sample origins—the “RunHarmony” function was utilized. Techniques such as principal component analysis (PCA) and t-distributed stochastic neighbor embedding (t-SNE) were harnessed to discern anchor points and to demarcate significant cellular clusters. By integrating the “FindNeighbors” and “FindClusters” functions at a resolution setting of 0.5, we delineated a total of 13 discrete cell clusters, which we then graphically represented on a “t-SNE” plot. Assignation of these clusters to predominant cell types was carried out manually in alignment with established marker genes. Additionally, the “COSG” R package, configured with parameters of mu equal to 10 and n_genes_user set to 100, facilitated the identification of distinctive markers for each of the cell groups.

### Assessment of different phenotype scores

Signature genes of various phenotypes (cholesterol efflux, lysosome, endoplasmic reticulum stress, angiogenesis, autophagy, acute inflammatory response, ferroptosis, and hypoxia) were collected from the Molecular Signatures Database (MSigDB) (13). The Single-Sample Gene Set Enrichment Analysis (ssGSEA) algorithm was then employed using default settings with the GSVA package to calculate phenotype-related scores for the chosen cell populations (14).

### DDR score calculation

201 DDR-related genes were extracted based on prior research (15). To assess each cell or sample’s DNA damage response (DDR) capacity, a pre-established computational approach was utilized. This method compensates for fluctuations in signal-to-noise ratios among different genes and cells by analyzing expression patterns of genes associated with DDR (16). Single cells had a threshold of 75% based on quartiles for DDR level, while bulk transcriptome data used the median value.

### Cell communication analysis

CellChat objects were established based on the UMI count matrix for each group (Normal and AD) using the “CellChat” R package (https://www.github.com/sqjin/CellChat) (17) and applied the “CellChatDB.human” ligand-receptor interaction database as reference data. Inter-cellular interaction studies employed standardized parameter configurations. Aggregated data from distinct cohorts were synthesized using the “mergeCellChat” utility, facilitating inter-group comparison in terms of interaction quantity and intensity. Visualization of divergences in interaction metrics among different cellular populations was achieved through the use of “netVisual_diffInteraction” and “netVisual_heatmap” tools. The principal communicating cells, both transmitting (origin) and accepting (target), were depicted by the “netAnalysis_signalingRole_scatter” feature. Finally, expression patterns for signaling genes across various categories were illustrated utilizing the “netVisual_bubble” function.

### WGCNA analysis

Utilizing the WGCNA module within R, we constructed a co-expression network for genes from the GSE65682 dataset, employing WGCNA as a biological method. The process entailed the following steps: utilizing the “goodSamplesGenes” function to filter out genes with missing data, visually selecting the most suitable soft threshold to calculate adjacency, transforming the gene expression matrix first into an adjacency matrix and subsequently into a topological overlap matrix (TOM) to delineate the genetic interactions within the network. By assessing differences in the TOM, we performed average linkage hierarchical clustering, followed by dynamic trimming of the resulting dendrogram to pinpoint modules of highly correlated genes. These module eigengenes (MEs) served as a proxy for the entirety of their respective gene modules. To correlate these MEs with clinical traits, Pearson correlation analysis was conducted. In the final stage, genes from modules with the strongest correlations to the DDR score were singled out for additional scrutiny.

### Development and validation of the risk scoring model

Univariate analysis was performed on intersecting genes to identify those with a statistically significant correlation to patients’ overall survival (OS) (P < 0.05). Subsequently, to refine the selection of genes and their corresponding risk coefficients significantly linked to survival outcomes, the LASSO (Least Absolute Shrinkage and Selection Operator) method was applied for Cox proportional hazards regression analysis, utilizing the “glmnet” package in the R statistical programming environment (18–20). Survival statistics were analyzed via Mantel-Cox tests, with the gene combination exhibiting the smallest P-value considered the final characteristic gene. Each patient with sepsis received a risk score based on coefficients derived from log-rank tests. The formula was presented as follows:


$$\text{riskScore}=\sum {\;}_{\text{i}}\text{Coefficient}s_{\text{i}}\times \text{Expression}\;\text{level}\;\text{of}\;\text{characteristics}\;\text{gene}{\text{s}}_{i}$$

Patients with sepsis were stratified into groups based on their risk levels, distinguishing between those with higher risk and those with lower risk, utilizing the median risk score as a discriminating factor. Survival trends were then plotted using the Kaplan-Meier method to aid in prognostication. The efficiency of the predictive model was assessed by examining the receiver operating characteristic (ROC) curves. To confirm the robustness of the signature’s prognostic capacity, its predictive performance was analyzed across four separate datasets by measuring the area under the curve (AUC).

### Evaluation of the prognostic model

We developed a predictive model that combines a risk assessment score with demographic variables, specifically age, and sex, to project the likelihood of patient survival over 28 days. We evaluated the accuracy of this predictive model through the use of calibration plots. Furthermore, we applied decision curve analysis to determine the practical advantage of using our model as opposed to relying solely on clinical features for prognostication.

### Enrichment analysis

Kyoto Encyclopedia of Genes and Genomes (KEGG) Gene Ontology (GO) enrichment analysis was performed using the previously described “clusterProfiler” R package (21). Gene ontology biological functions include biological processes (BP), molecular functions (MF), and cellular components (CC). P-values less than 0.05 were considered statistically significant.

Utilizing the Gene Set Variation Analysis (GSVA) approach, we analyzed the variability of biological process heterogeneity and pathway function using the “GSVA” software tool in R (14). For this analysis, we employed the “c5.go.bp.v7.5.1.symbols” Hallmark gene sets sourced from the MSigDB compendium. These gene sets were selected as the optimal representatives for GSVA application. We identified unique molecular signatures within DDR categories and quantified the disparities across biological operations and signal transduction processes with the aid of the “limma” software tool in R. We considered absolute t-values combined with GSVA scores exceeding 2 to reflect statistical significance.

In parallel, gene set enrichment analysis (GSEA) was conducted using the “clusterProfiler” toolkit in R, aiming to discern variations in pathway activities. The results were prioritized based on Normalized Enrichment Scores (NES), and we adopted a p-value threshold of less than 0.05 to ascertain statistical significance. Furthermore, we appraised the classical disease-relevant signaling pathway activity differences between our study cohorts utilizing the progeny R package, with p-values lower than 0.05 denoting statistical significance.

### Sepsis immunity

The ssGSEA algorithms using the GSVA package (14) were applied to examine immune infiltration levels. Briefly, the proportions of different immune cells present in each specimen were evaluated utilizing universal marker genes. These proportions were then quantified by estimating the fractional presence or the comparative prevalence of every immune cell type through the application of the referenced computational approaches. The Wilcoxon rank-sum method was utilized to ascertain the disparity in immune cell infiltration amongst distinct cohorts. To depict the various extents of immune infiltration in Alzheimer’s disease specimens based on discrete methodologies, a heatmap was generated. Additionally, the “ESTIMATE” software, accessible in R, was harnessed to deduce the levels of immune infiltration in individuals with sepsis. Immune checkpoints, which are pivotal in curtailing overt immune responses, comprise a range of molecules—including those involved in antigen display, cellular adhesion, co-inhibition, and co-stimulation, as well as ligands and receptors—found on immune cells that modulate the intensity of immune responses. The gene expression of renowned immune checkpoints was contrasted between the cohorts in question.

### Modeling sepsis and peripheral blood sample collection in rats

To validate DDR-associated target genes *in vivo*, a rat model of sepsis was established. In this research, male Sprague-Dawley rats that tipped the scales at 250 to 300 grams were used. They were kept in an environment where both temperature and humidity were regulated. The facility provided a consistent cycle of 12 hours of light followed by 12 hours of darkness. Additionally, the rats had unrestricted access to both food and water. All experimental protocols were approved by the Fujian Medical University Institutional Animal Care and Use Committee.

The sepsis model was established through the administration of cecal ligation and puncture (CLP), in line with methods outlined in the earlier literature (22–24). Anesthesia was induced in the rats using sodium pentobarbital, which was delivered via intraperitoneal injection at a dosage of 50 mg/kg. Under aseptic conditions, a midline laparotomy was performed to expose the cecum and surrounding intestines. A 3-0 silk suture was utilized to ligate the cecum beneath the ileocecal valve while preserving the integrity of the intestinal tract. An 18-gauge needle was used to puncture the cecum twice, facilitating the expulsion of a minor quantity of fecal content. Subsequently, the cecum was repositioned within the abdominal cavity, and the open wound was closed in a stratified manner. Sham-operated rats underwent the same surgical procedure without cecal ligation and puncture. Postoperatively, animals received subcutaneous injections of pre-warmed (37°C) sterile 0.9% saline (5ml/100g) for fluid resuscitation.

For peripheral blood sampling, rats were subjected to intracardiac puncture under deep sedation with pentobarbital sodium (50mg/kg, i.p.) 24 hours after CLP or sham surgeries. Blood samples were drawn into tubes containing ethylenediaminetetraacetic acid (EDTA) and subsequently, the plasma was isolated using a centrifugal process at 2,000 times gravity for a quarter-hour duration, maintained at a temperature of 4°C. For subsequent examinations, these plasma specimens were preserved at a temperature of -80°C.

### Generation of adenovirus-mediated ARL4C knockdown rat model via intravenous injection

To confirm the involvement of ARL4C in sepsis further, a model with reduced ARL4C expression was developed in an *in vivo* setting. In summary, adenoviral vectors containing shRNA sequences specifically targeting the ARL4C gene in rats (Ad-shARL4C) alongside a non-targeting control shRNA sequence (Ad-shNC), both sourced from RiboBio in Guangzhou, China, were utilized. For each subject in the experimental group, around 30 billion PFU of Ad-shARL4C in 200 μL saline were administered via tail vein injection. Contrarily, the control group received a similar dose of Ad-shNC. Evaluation of the gene silencing efficacy was carried out on the 14th day post-injection using qRT-PCR. RNA from blood samples was isolated using Trizol reagent (Invitrogen, USA). These samples then underwent qRT-PCR with primers specific to rat ARL4C to measure its expression levels. Additionally, sepsis was induced in the animal model on the same day, followed by blood collection for analysis using the previously outlined method.

### Validation of ARL4C *in vitro*

The RAW264.7 cells, a mouse macrophage cell line, were obtained from Beyotime Biotechnology. They were cultured in a complete medium consisting of high glucose DMEM (Gibco), 10% fetal bovine serum (FBS, Gibco), 100 U/mL penicillin, and 100 mg/mL streptomycin, and maintained at 37°C with 5% CO2. Subsequently, the RAW264.7 macrophages were exposed to LPS (1 μg/mL) in DMEM without FBS following preincubation in a complete growth medium. For knockdown experiments, shRNA plasmids targeting ARL4C and negative control shRNA were procured from RiboBio (Guangzhou, China), and the specified shRNA lentiviral vectors were generated in 293 T cells. Upon transfection of RAW264.7 cells with the lentivirus, they were selected using 2 μg/mL puromycin for 72 h.

### Flow cytometry analysis

A total of 1*10^6^ transfected cell cells were prepared in six-well plates for the flow cytometry assay, following the standard Annexin V-FITC/PI double staining kit protocol (Invitrogen). Subsequently, the samples were analyzed using a flow cytometer (BD Biosciences, Franklin Lakes, NJ, USA) after incubation in the dark for 15 minutes. Additionally, the levels of reactive oxygen species (ROS) in the cells were measured. The cells were treated with H2DCF-DA for 1 hour, then harvested with PBS, and the ROS levels were assessed using flow cytometry (BD Biosciences, Franklin Lakes, NJ, USA). The experiment involved three biological replicates.

### RT-qPCR

Peripheral blood samples were used to extract total RNA utilizing the Trizol reagent (Life Technologies, USA), which was subsequently reverse transcribed into complementary DNA with the RevertAid First Strand cDNA Synthesis Kit, following the manufacturer’s guidelines. The ABI PRISM 7500 real-time PCR system (Applied Biosystems, USA) was employed for quantitative RT-PCR, utilizing the SYBR Premix EX Taq (Takara, Japan). The primer sets were demonstrated as follows:

CD247
Forward: 5’- GAGTGGTCTGGTGGCTGAAAT -3’
Reverse: 5’- GTTCCAACTGCCACACTTCTGA -3’
RPL7
Forward: 5’- GAGAAGGTGCTGATGACTTGGA -3’
Reverse: 5’- TCTTGGACCTTCTTGGCTTCA -3’
RPL31
Forward: 5’- ATGGCTGAGAAGCGCAACTA -3’
Reverse: 5’- GCAGTAATCCAGCACCAGCA -3’
ARL4C
Forward: 5’-CGGAGTGACATCTGGATATGC-3’
Reverse: 5’-GCCTCAACTTCGTATAGACTT-3’

To assess the relative mRNA expression quantification, the target gene’s cycle threshold (CT) was compared to the β-actin CT value, and the data was represented as fold changes through the 2-ΔΔ Ct technique.

### Enzyme-linked immunosorbent assay

Rat peripheral blood samples were collected and subjected to the measurement of IL-1β, IL-10, TNF-α, and IL-18 using ELISA kits following the manufacturer’s instructions (R&D Systems, USA). Briefly, blood samples were centrifuged at 2000g for 10 minutes and the supernatants were harvested for further testing. Reagent diluent was added to each microplate well, followed by the addition of the sample or standard. The plates were securely covered and incubated for 2 hours at room temperature. Following incubation, the solution in each well was aspirated and washed thrice. Subsequent to the initial rinse cycle, a volume of 100 microliters of the conjugated solution was carefully introduced into every individual well. These were then shielded and allowed to stabilize for an additional duration of two hours at an ambient thermal condition. Upon the conclusion of this second incubation phase, a repeat of the aspiration and cleansing process ensued. Following this, wells were each infused with 100 microliters of the specified Substrate Solution, and the entire assemblage was then left to incubate for 20 minutes. To halt the enzymatic reaction, a measure of 50 microliters of Stop Solution was promptly dispensed into each well. Optical density determinations were subsequently performed with a spectrophotometer calibrated to 450 nanometers. Utilizing established standard calibration curves, the levels of interleukins IL-1β and IL-10, in conjunction with tumor necrosis factor-alpha (TNF-α) and IL-18, were meticulously computed.

### Statistical analysis

The R program was employed for the analysis of all collected data and statistics. We compared the survival probabilities of the distinct cohorts by applying Kaplan-Meier estimations in conjunction with log-rank examinations. For the illustration of survival distributions, we engaged the ggsurvplot package from within R. Evaluation of prognostic elements was undertaken through a univariate Cox regression approach, while a Lasso-based Cox analysis isolated significant influential factors on patient outcomes. For graphing purposes, the “ggplot2” toolkit in R was utilized, and survival durations, measured as overall survival (OS), were computed using the survival package.

The association between two metric variables was investigated via Spearman’s rank correlation. When examining differences in metric variables between cohorts, we utilized either the Wilcoxon rank-sum assessment or the two-sided t-test, depending on the data distribution. To assess disparities in categorical data across the groups, the chi-squared method was applied. All statistical procedures were executed within the confines of R. A P-value threshold of less than 0.05 was predetermined to denote statistical significance.

## Result

### Single-cell analysis of the DDR in sepsis has revealed varying levels of DDR activity that correlate with different prognoses in sepsis patients

**Figure 1:**
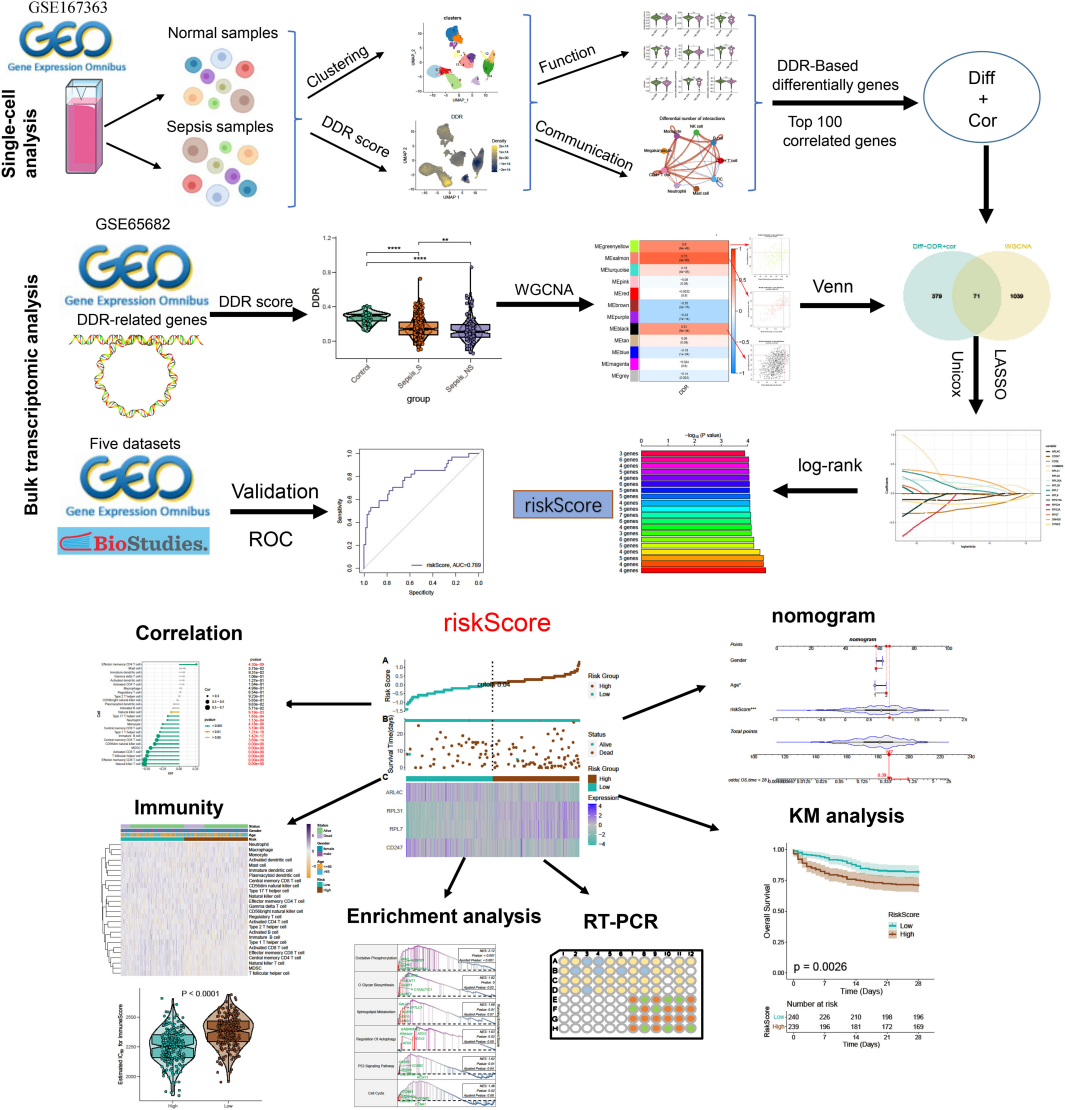
The flowchart of this study. **p < 0.01, ****p < 0.0001.

**Figure 2:**
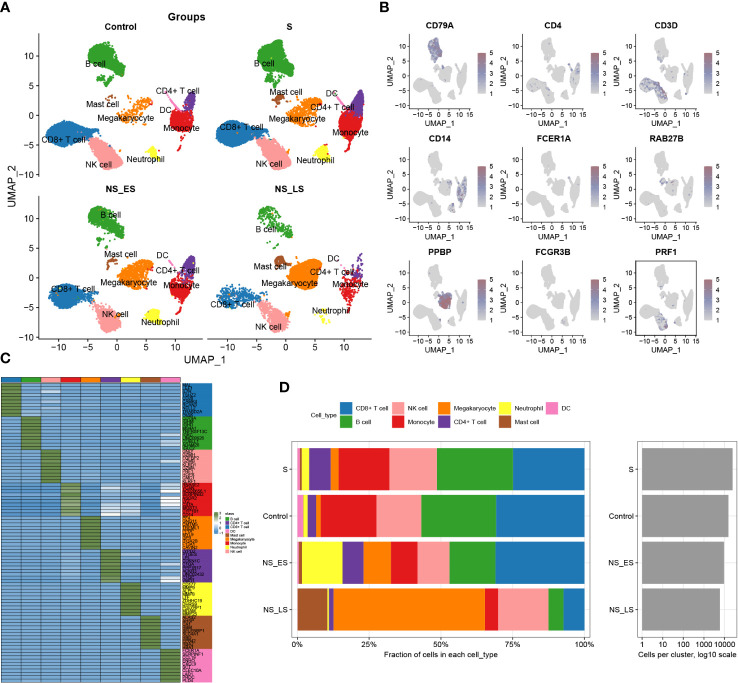
Annotation of single-cell data. **(A)** The UMAP projection of single cells from GSE167363, showing the distribution of 9 cell subtypes in each group. **(B)** Distinctive marker genes for each cell subtype. **(C)** A heatmap displayed the distribution of the top 10 differentially expressed genes specific to different cell subtypes. **(D)** Cell type fractions of each sample and group.

The study’s flowchart is displayed in **Figure 1**. To explore the variation in DDR activity among infiltrated immune cells during sepsis, we conducted a comprehensive analysis of publicly available single-cell sequencing data associated with sepsis. After conducting a quality check, a total of 55268 high-quality cells (39859 in sepsis samples and 15409 in healthy control samples) were identified as suitable for further study. To analyze the cellular characteristics of sepsis with varying degrees of severity, we categorized the cells in the sepsis group according to patient prognosis into several groups: Control (15409 cells), survival (S, 24464 cells), non-survival early stage (NS_ES, 9480 cells), and non-survival late stage (NS_LS, 5915 cells), and T0 or T6 represents the time of blood collection after the diagnosis of the disease. **Supplementary Figure S1A** exhibited the nFeature_RNA and nCount_RNA expressed per cell in all samples, indicating cell samples used in the study were of high quality. The distribution of each group of cells is presented in **Supplementary Figures S1B, C**. The cells were grouped into 14 clusters and immune cell subtypes were identified (**Figures S1C, D**). These include B cells (n=12323) expressing CD79A, CD4+ T cells (n=3092) marked by CD4, CD8+ T cells (n=14217) expressing CD3D, Monocytes (n=8473) marked by CD14, DCs (n=556) identified by FECR1A, Mast cells (n=907) positive for RAB27B, Megakaryocytes (n=4940) defined by their classical marker PPBP, Neutrophils (n=2216) marked by FCGR3B, and NK cells (n=2216) marked by PRF1 (**Figures 2A, B**). In addition, for each cellular cluster, the ten most characteristic genes were displayed using a heatmap (as seen in **Figure 2C**). The proportion of different cell types present in each sample and collective grouping can be observed in **Figure 2D**. In brief, megakaryocytes, dendritic cells (DC), and mast cells were predominantly enriching in the NS_LS group, high percentages of CD8+ T cell, B cell, and neutrophils were found in the NS_ES group, and high levels of monocyte, B cell, NK cell, and CD8+ T cell were observed in the S group. Moreover, a reduction in the proportion of CD4+ T cells, CD8+ T cells, and neutrophils was observed in the NS_LS group. Interestingly, we observed that as the prognosis of sepsis deteriorated, the proportion of megakaryocytes among immune cells exhibited a progressive increase, suggesting their potential pivotal role in the exacerbation of sepsis. Additional research is warranted to investigate how these cells might contribute to the pathogenesis of DNA damage associated with sepsis.

**Figure 3:**
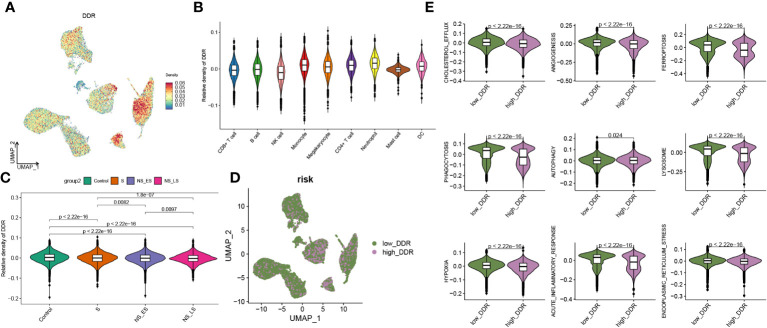
Features of DDR at the single-cell level. **(A)** An UMAP plot presented the difference of DDR density in GSE167363 (The relative density plots, constructed based on the magnitude of the DDR scores, demonstrate that a higher density of DDR scores is indicative of a high DDR score within that cellular category.) of cell subtypes based on the UCell algorithm. **(B)** Violin diagram exhibited the differences in GM score among each cell types. **(C)** Relative score of DDR in each group. **(D)** UMAP plot demonstrated the difference in the distribution of high and low DDR group at the single-cell level in sepsis samples, bounded by the 75% quantile of the DDR score. **(E)** Classic phenotype-related scores (The classical biofunctional phenotypic score was calculated using ssGSEA, and the score level represented the degree of functional enrichment) in each group.

Additionally, we evaluated the DDR score in each group and found a higher level of DDR in sepsis samples, particularly in survival groups (**Supplementary Figure S2A**). A 201-DDR-related gene-based score for each cell subtype was calculated using the ‘UCell’ algorithm to clarify the DDR degree in immune cells in GSE167363, without distinguishing between groups. Monocytes, megakaryocytes, CD4+ T cells, and neutrophils have a comparatively higher DDR score than other cells, as illustrated in **Figures 3A, B**. In addition, sepsis samples had lower scores than the control samples, and both the NS_LS and NS_ES groups had lower DDR scores than the S group, while the NS_ES group had the lowest DDR score (**Figure 3C**), revealing that low DDR score was intensively associated with the poor prognosis of sepsis. Subsequently, cells in sepsis samples were divided into low- and high-DDR groups based on the 75% quantile of DDR score (**Figure 3D**), and the classic phenotypic (Cholesterol_efflux, angiogenesis, ferroptosis, phagocytosis, autophagy, lysosome, hypoxia, acute inflammatory response, and endoplasmic reticulum stress) scores in each group were further computed and compared (**Figure 3E**). The low-DDR group exhibited elevated scores across all phenotypic categories (as illustrated in **Figure 3E**). Interestingly, an analysis of the cellular composition for each cohort showed a reduction in the proportion of every cell type (CD8+ T cell, B cell, NK cell, monocyte, megakaryocyte, CD4+ t cell, neutrophil, mast cell, and DC) in high DDR group (refer to **Supplementary Figure S2B**). Among these cells, the most notable decreases in proportion within the high DDR group were observed in mast cells and dendritic cells.

In this section, we found higher DDR levels in sepsis than in controls, with CD4+ T cells, monocytes, megakaryocytes, and neutrophils showing the highest DDR, and observed an association between lower DDR scores and poorer sepsis prognosis.

### A strong correlation was found between increased levels of DDR and heightened intercellular communication in sepsis

**Figure 4:**
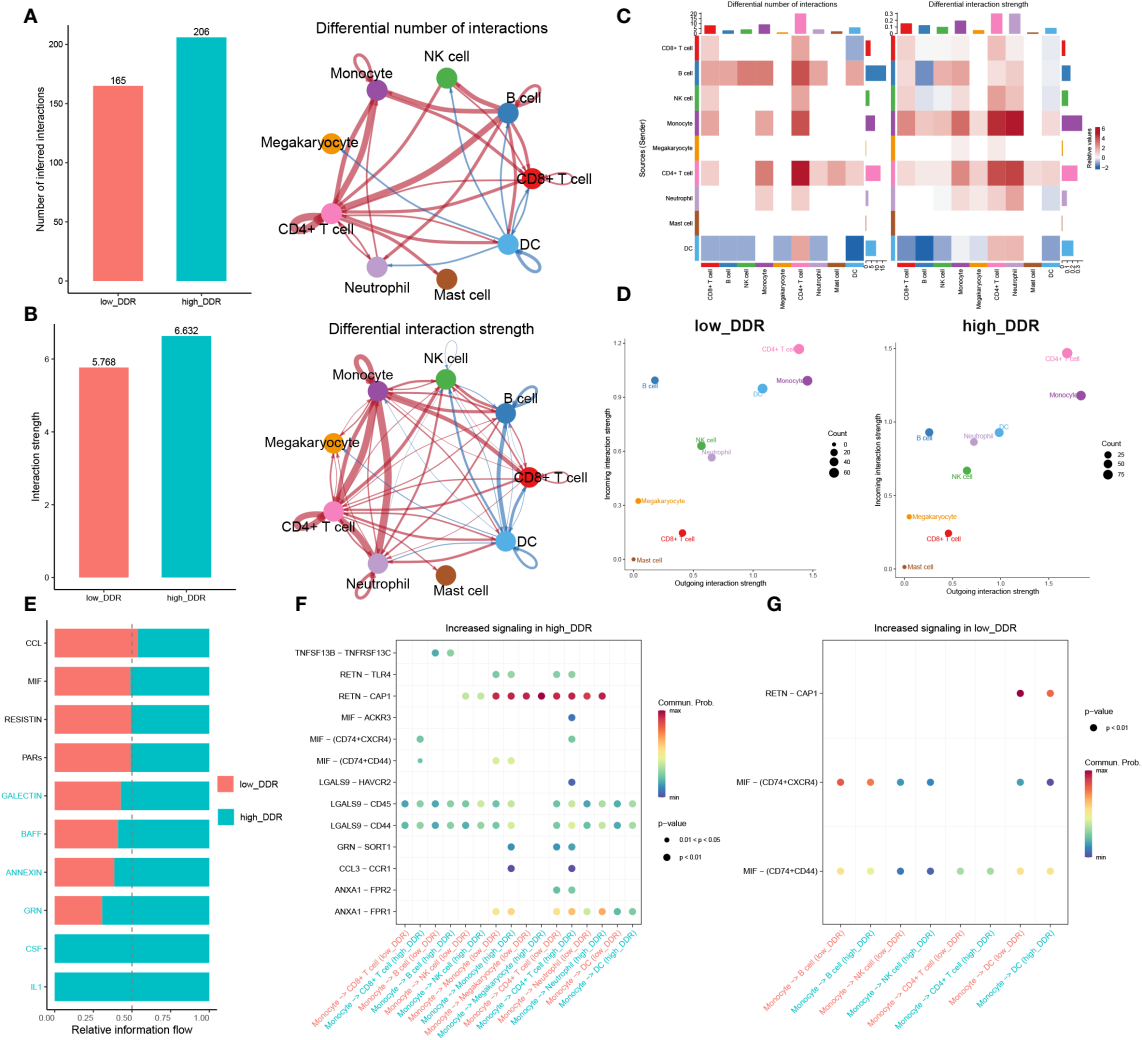
Intercellular communication analysis. **(A, B)** Charts with bars and circles depicting the variations between low-DDR and high-DDR groups in the quantity of interactions **(A)** or strength of interactions **(B)** in the network of cell-cell communication. Stronger interactions are represented by thicker lines, and increased or decreased signaling in the high-DDR group when compared to the low-DDR group was represented by red or blue colors, respectively. **(C)**Heat-maps exhibiting the differential number or strength of interaction. **(D)** The incoming and outgoing interaction strength of each cell type in the low- and high-DDR groups. The strength of incoming or outgoing interactions can be described as the probability of signaling to or from a population of cells during communicative events. **(E)**The variations in intercellular signaling networks between the high- and low-DDR groups. **(F, G)** Dot plot depicting the difference in signaling molecules from monocyte cells to other immune cells between low- and high-DDR groups.

To investigate DDR-related intercellular communication during sepsis, we analyzed Cell-Chat Ligand-Receptor (LR) interactions. Our findings revealed that as DDR levels increased, both the number and strength of interactions also increased. In the high-DDR group, CD4+ T cells and CD8+ T cells showed stronger interactions with most cell types compared to the low-DDR group. Additionally, monocytes had extensive intercellular communication with CD4+ T cells, CD8+ T cells, and neutrophils in the high-DDR group, whereas in the low DDR group, DCs communicated with B cells and NK cells frequently (**Figures 4A–C**). The strength of incoming or outgoing interactions can be described as the probability of signaling to or from a population of cells during communicative events. Furthermore, the strengths of outgoing and incoming interactions of cells in two groups were examined. In brief, monocytes, CD4+ T cells, and DC displayed strong outgoing and incoming interaction capabilities in both low- and high-DDR groups, while B cells showed intensive incoming interaction strength in these two groups (**Figure 4D**). Among them, DC exhibited strong outgoing and incoming interaction capabilities, especially in the low-DDR group. Those findings suggested that immune cells especially monocytes and CD4+ T cells may play a crucial role in the progression of sepsis, particularly in cases with high levels of DDR. DC may serve as a crucial element for patients exhibiting minimal DDR levels. To discern the distinct biochemical communication routes among the cohorts, the intensities of their interactions were analyzed. The high-DDR group exhibited higher activity in signaling pathways such as GALECTIN, BAFF, ANNEXIN, GRN, CSF, and IL-1 compared to the other group. Notably, only the high-DDR group exhibited activity in CSF and IL-1 (**Figure 4E**). We next further explored the significant ligand-receptor pairs between monocytes and other cell types. In the high-DDR group, monocytes up-regulated LGALS9-CD44/CD45 and RETN-CAP1 to interact with the majority of cells, whereas in the low-DDR group, MIF-(CD74+CXCR4)/(CD74+CD44) was up-regulated for the same purpose (**Figures 4F, G**).

In this section, we found that strong correlation between increased levels of DDR and heightened intercellular communication, implicating enhanced interactions, particularly among monocytes, CD4+ T cells, and DCs, as potential contributors to the severity of sepsis. With high DDR, there is an upregulation in signaling pathways and ligand-receptor pairs that intensify communication, suggesting these cells play a critical role in the DDR-related progression of sepsis.

### Investigation of DDR-related hallmark genes in sepsis

**Figure 5:**
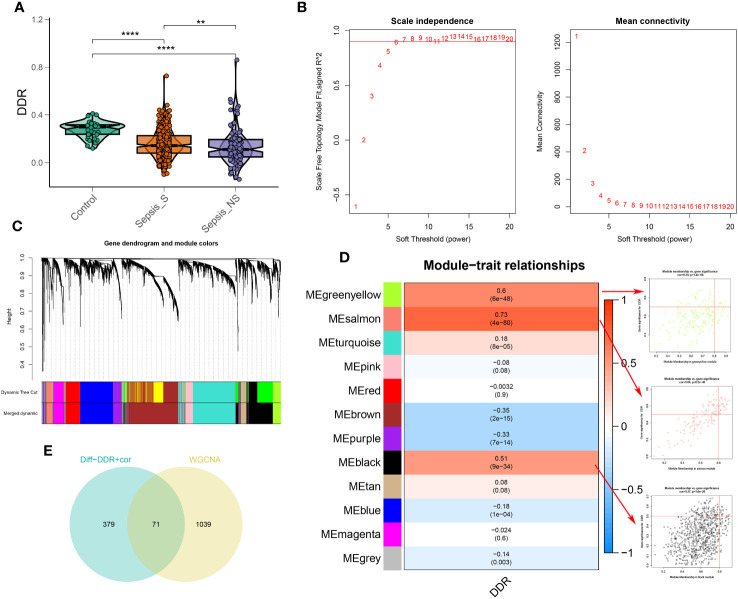
Identification of DDR-associated genes. **(A)** DDR score established via the ssGSEA algorithm to compare differences in DDR levels between the control, sepsis-survival and sepsis-non-survival group in the GSE65682 dataset. **(B)** The selection of soft threshold power of WGCNA. **(C)** Dendrogram of co-expression module clustering. **(D)** The WGCNA analysis investigated the modules that were most closely related to the DDR score. Scatter plots represented the module membership (Greenyellow, salmon or black) and gene significance of GM. **(E)** Interaction of DDR-associated genes screened from WGCNA and single cell technology. **p < 0.01, ****p < 0.0001.

To explore the association between DDR and sepsis, we applied the single sample Gene Set Enrichment Analysis (ssGSEA) algorithm to compute DDR scores across diverse patient cohorts using the bulk sepsis transcriptome dataset GSE65682, which comprises 42 healthy and 760 sepsis samples (479 survival, 281 no survival). The analysis demonstrated that the control cohort exhibited elevated DDR scores relative to the sepsis cohort, and the non-survival (sepsis_NS) cohort presented the lowest DDR scores, corroborating the observations from single-cell analyses (**Figure 5A**). Subsequently, WGCNA was employed to elucidate the regulatory patterns associated with DDR and to validate DDR-related genes through their expression profiles in the dataset GSE65682. Employing an optimal soft-thresholding power (β=6), we conducted a hierarchical clustering analysis of the sample data, discerning 12 gene co-expression modules, designated by unique colors in the dendrogram (**Figures 5B–D**). Of these clusters, the black (R=0.51), salmon (R=0.73), and green-yellow (R=0.6) modules showed robust associations with DDR, leading to the selection of 1,110 genes for further scrutiny. In the ensuing step, we refined our focus to 71 genes by intersecting the 450 genes most indicative of DDR dynamics from the single-cell study (merging differentially expressed genes from high and low DDR groups with the top 100 DDR-correlated genes in single cells) with the three modules displaying the strongest DDR associations from WGCNA (**Supplementary Figure 5E**).

Moreover, our functional enrichment analysis ascertained the significant representation of DDR-characteristic genes within intracellular protein transportation, modification, and anchoring pathways (**Supplementary Figure S3A**). Furthermore, the Kyoto Encyclopedia of Genes and Genomes (KEGG) pathway analysis implicated these genes across various biological pathways, including those related to coronavirus disease (COVID-19), ribosomal function, T-helper cell differentiation (Th1 and Th2), primary immunodeficiency disorders, and the hypoxia-inducible factor-1 (HIF-1) signaling pathway (**Figure S3B**).

In this section, we analyzed the relationship between DDR and sepsis using bulk sepsis transcriptome data. The ssGSEA algorithm revealed that healthy subjects had higher DDR scores than sepsis patients, with the lowest scores found in non-surviving sepsis patients. Through WGCNA, we identified 71 genes significantly associated with DDR. Based on these genes, we will construct a DDR-related risk score model in the subsequent section.

### Construction of a DDR-based riskScore system

**Figure 6:**
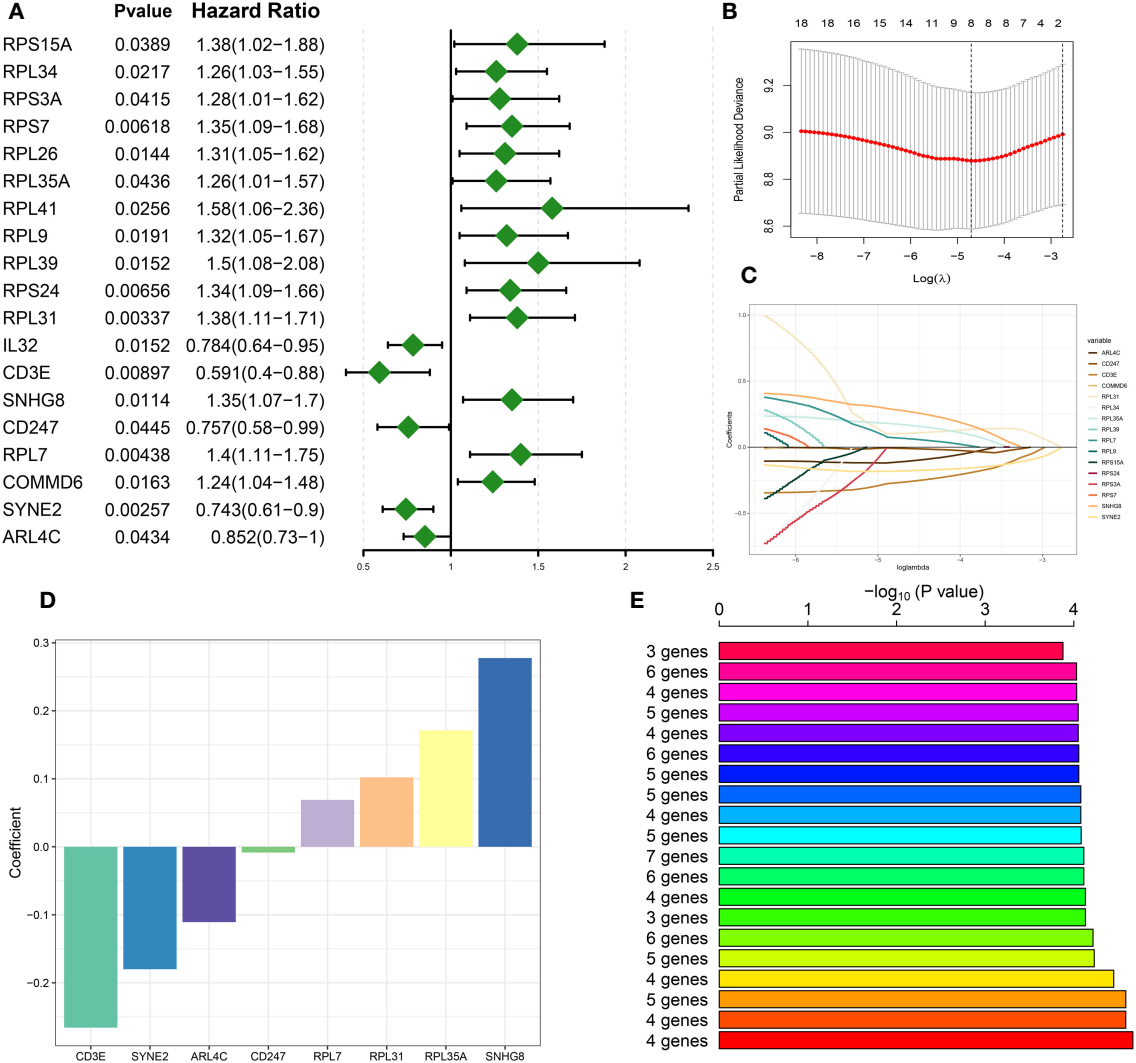
Construction of DDR-based riskScore system. **(A)** Univariate cox analysis on the intersection genes. **(B)** Tuning feature selection in the LASSO model. **(C)** LASSO coefficient profiles of the DDR-related characteristic genes. **(D)** The specific coefficient value of the 4 Genes associated with GM identified by the optimal lambda value. **(E)** Kaplan-Meier analysis of 2^8-1 = 255 gene combinations, the top 20 signatures were ranked and the signature comprising four genes was selected due to its relatively large negative logarithm (-log10) of the p-value combined with a minimal gene count.

Utilizing preceding analyses, including single-cell, intercellular communication, enrichment, and characteristic gene selection, this study has elucidated DDR’s crucial role in the pathogenesis and advancement of sepsis, pinpointing pertinent immune cells, signaling pathways, and molecular targets. The subsequent phase of our research will involve the systematic construction of a risk prediction model related to DDR in sepsis. This model aims to integrate risk assessment with tailored therapeutic approaches. Based on the 71 DNA damage response (DDR)-related genes, univariate Cox proportional hazards analysis was conducted, yielding 19 genes that exhibited a statistically significant correlation with patients’ in the bulk sepsis transcriptome data GSE65682 overall survival (Displayed as a univariate analysis hazard ratio [HR]) (**Figure 6A**). Subsequently, least absolute shrinkage and selection operator (LASSO) Cox regression analysis and log-rank (Mantel-Cox) tests were utilized to refine the identification of genes associated with survival outcomes (**Figures 6B–E**). Finally, we acquired 4 feature genes (ARL4C, CD247, RPL7, and RPL31), and the DDR-related riskScore model consisting of 4 genes was constructed. The formula was presented as follows: riskScore = (-0.116217907 × ARL4C) + (-0.012432987 × CD247) + (0.065124879 × RPL7) + (0.102865839 × RPL31). Based on the median riskScore, patients from bulk sepsis transcriptome data were divided into high- and low-risk categories.

### The evaluation of the DDR-based RiskScore system demonstrated its efficacy in prognosticating sepsis outcomes

**Figure 7:**
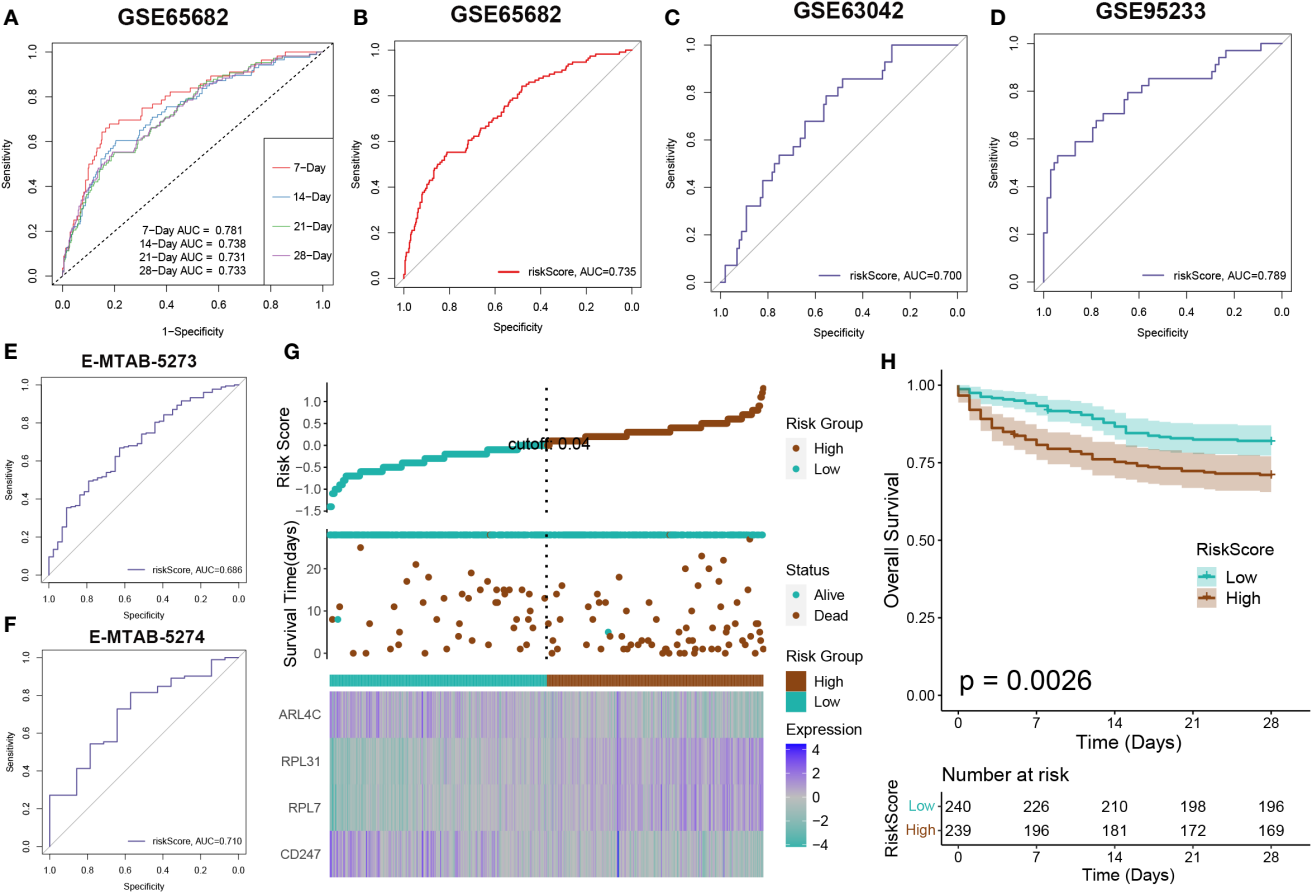
Risk model evaluation. The ROC curve was used to evaluate the performance of the model in the GSE65682 **(A, B)**, GSE63602**(C)**, GSE95233 **(D)**, E-MTAB-5273 **(E)**, and E-MTAB-5274. **(F)** datasets. **(G)** The distribution of the riskscore, patients’ survival status as well as gene expression signature in the combination dateset. **(H)** Overall survival situation between the low- and high-risk group.

Survival analysis was applied to assess the effectiveness and consistency of the riskScore-based prognostic prediction model. The AUC values for 7, 14, 21, and 28-day mortality in GSE65682 were all greater than 0.7 (**Figure 7A**). Furthermore, the riskScore-based AUC values for 28-day mortality in five datasets (GSE65682, GSE63042, GSE95233, E-MTAB-5273, and E-MTAB-5274) were all greater than 0.6, indicating the high prediction accuracy of the riskScore (**Figures 7B–F**). Subsequently, sepsis samples were classified into high- and low-risk categories. Patients in the high-risk category tended to experience reduced mean survival periods, frequently succumbing during the illness’s initial phase. Individuals within the low-risk bracket exhibited increased expression of ARL4C and CD247. On the other hand, those at higher risk were characterized by elevated levels of RPL31 and RPL7, as depicted in **Figure 7G**. Furthermore, survival analysis indicated that low-risk patients, who presented with widespread expression of ARL4C and CD247, maintained enhanced survival probabilities compared to their high-risk counterparts, as illustrated in **Figure 7H**.

**Figure 8:**
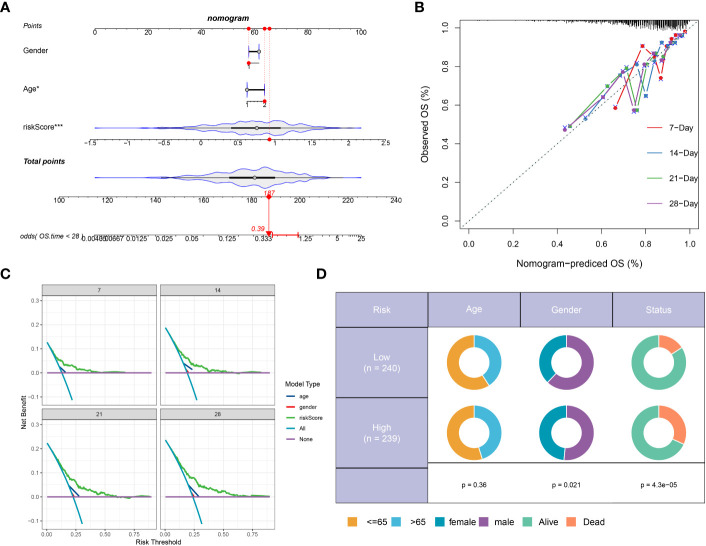
Construction and validation of a prognostic prediction model based on the riskScore. **(A)** Construction of a nomogram based on riskScore and clinical characteristics in the combination dateset. **(B)** Correction of the characteristic curve based on riskscore and pathological characteristic. **(C)** DCA indicating the clinical benefit of the nomogram. **(D)** The distribution of clinical features and survival status in the low- and high-risk groups.

We developed a prognostic nomogram for sepsis that incorporates demographic factors such as patient age and sex along with a risk score. Each predictor within the nomogram was assigned a specific number of points, and the sum of these points across all predictors provided a cumulative score indicative of the probability of an adverse outcome in sepsis. This cumulative score is visually represented in **Figure 8A**. The predictive accuracy of our nomogram was confirmed through a calibration plot, depicted in **Figure 8B**. Decision curve analysis (DCA) further demonstrated the clinical utility of our nomogram, which is grounded on the computed risk score (**Figure 8C**). Additionally, we provided a schematic representation of the demographic distribution by age sex, and survival statuses, stratified according to two risk categories. This analysis did not reveal a significant difference in the age distribution across cohorts, but it did show a higher incidence of males in the low-risk category compared to the high risk, suggesting that sex may play a significant role in the prognosis of sepsis (**Figure 8D**).

In this section, we assessed a riskScore-based model’s effectiveness in predicting sepsis mortality. Survival analysis categorised sepsis patients into high or low risk, with gene expression differences observed in each group. A prognostic nomogram, considering age, sex, and risk score, showed good predictive accuracy and clinical utility for sepsis outcomes.

### Enrichment analysis of DDR-based riskScore system in sepsis

**Figure 9:**
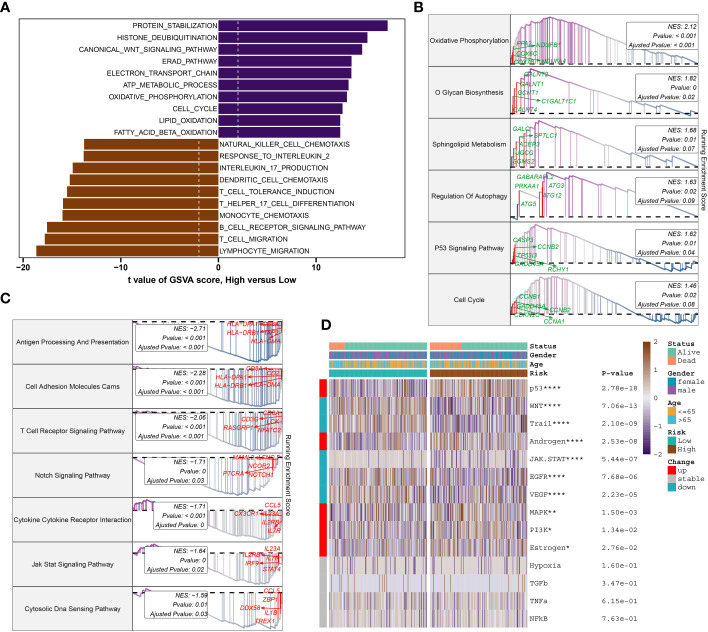
Molecular characteristic and functional annotation of the DDR-riskScore model in sepsis. **(A)** The GSVA identified significant differences in biological functions between the high- and low-risk groups. Positive values indicate that the biological function is enriched in the high-risk group, while negative values indicate that the biological function is enriched in the low-risk group. **(B)** Top five up-regulated pathways in the high-risk group. **(C)** Top five pathways down-regulated in the high-risk group. **(D)** Heatmap displaying the difference of pathogenic pathways in sepsis patients at low and high risk. Age, gender, and survival status are displayed as patient annotations. *p < 0.05, **p < 0.01, ****p < 0.0001.

To further elucidate the DDR-related mechanisms in sepsis, feature genes of the high- and low-risk group in GSE65682 were explored. 237 genes were down-regulated while 417 genes were up-regulate, and finally we obtained the top 5 up-regulate genes (ENY2, EAF2, LSM3, C14orf2, and COX7B) and top 5 down-regulate gens (CCL5, CD2, TBX21, TRD, and CD3E) (**Supplementary Figures S4A, B**). Based on these feature genes, we annotated the enriched functional characteristics using Gene Set Variation Analysis (GSVA) and Gene Set Enrichment Analysis (GSEA). The two groups had distinct pathway enrichment patterns. In the low-risk group, immune response-related biological functions were markedly enriched. These functions encompassed natural killer cell chemotaxis, response to interleukin-2, production of interleukin-17, dendritic cell chemotaxis, induction of T cell tolerance, T cell and lymphocyte migration, T helper 17 cell differentiation, monocyte chemotaxis, B cell receptor signaling pathway, among others. In the high-risk group, the GSVA has identified a predominant involvement of several biological functions that play critical roles in cellular and molecular processes. These functions include protein stabilization, histone deubiquitination, canonical Wnt signaling pathway, Reed-Sternberg cell pathway (implied by the context to mean “Reed pathway”), electron transport chain, ATP metabolic process, oxidative phosphorylation, cell cycle, lipid oxidation, and fatty acid β-oxidation (**Figure 9A**). Next, the top 5 up-regulated pathways (Oxidative Phosphorylation, O Glycan Biosynthesis, Sphingolipid Metabolism, Regulation of Autophagy, and P53 Signaling Pathway) and top 5 down-regulated pathways (Antigen Processing And Presentation, Cell Adhesion Molecules Cams, T Cell Receptor Signaling Pathway, Notch Signaling Pathway, and Cytokine Cytokine Receptor Interaction) of the high-risk were obtained via GSEA (**Figures 9B, C**). Additionally, Pathogenic pathways vary significantly among patients with different risks for sepsis. High-risk patients display marked activity in pathways such as P53, androgen, MAPK, PI3K, and Estrogen. Conversely, low-risk patients exhibit excessive activation of WNT, Trail, JAK-STAT, EGFR, and VEGF compared to high-risk patients with sepsis (**Figure 9D**).

Gene analysis revealed distinct up-regulated and down-regulated genes and pathways between high- and low-risk groups, suggesting varied mechanisms driving sepsis in each group.

### Immune characteristics analysis of DDR-based riskScore system in sepsis

**Figure 10:**
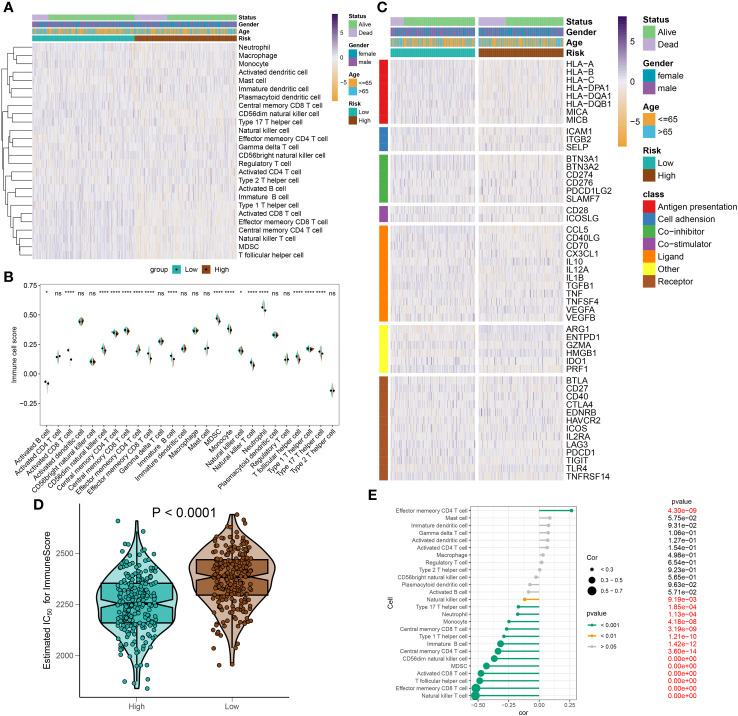
Immunological features of sepsis patients at low and high risk. **(A)** The heatmap showing the degree of infiltration of 28 immune cell subtypes in high- and low-risk groups. **(B)** Differences in immune cell scores between high- and low-risk groups. **(C)** Heatmap depicting the differences in immune-modulators and patients’ survival status between high- and low-risk groups. **(D)** A comparison of the immuneScore between high- and low-risk groups. **(E)** The interaction between riskScore and 28 immune cell subtypes. *p < 0.05, ****p < 0.0001. NS, no significant difference.

To elucidate immune cell infiltration patterns in high- and low-risk groups, we compared 28 immune cell subtypes using single-sample gene set enrichment analysis (ssGSEA) to calculate their respective scores (**Figure 10A**). The low-risk group demonstrated enhanced levels of various immune cells, including activated B cells, CD4+ and CD8+ T cells, CD56^bright natural killer (NK) cells, central memory T cells, γδ T cells, immature B cells, monocytes, neutrophils, follicular helper T cells (Tfh), and Th17 cells. Conversely, the high-risk category only exceeded the low-risk group in effector memory CD4+ T cell scores (**Figure 10B**). Further investigation of immune modulators in both groups revealed distinct immunological profiles in individuals with sepsis (**Figure 10C**). Genes involved in antigen processing (HLA-A, HLA-B, HLA-C, HLA-DPA1, HLA-DQB1, MICA, MICB), cell adhesion (ICAM1, ITGB2), inhibitory (BTN3A1, BTN3A2, SLAMF7), and stimulatory (CD28, ICOSLG) signals, as well as ligands and receptors (CCL5, CD40LG, CD70, CX3CL1, IL12A, IL1B, TGFB1, VEGFB, CD27, CTLA4, ICOS, IL2RA, LAG3, PDCD1, TIGIT, TNFRSF14), were mostly upregulated in low-risk individuals. In contrast, high-risk individuals predominantly showed upregulation of the VEGFA ligand and the TLR4 receptor (**Supplementary Figures S5A–F**). Additionally, the comparative analysis of immune scores revealed superior immune profiles in the low-risk group than in their high-risk counterparts (**Figure 10D**), with a negative correlation between risk score and immune cell types across the board, denoting stronger immune infiltration (**Figure 10E**).

In this section, we have illustrated the patterns of immune cell infiltration in high- and low-risk groups and identified potential targets for immunotherapy in sepsis patients stratified by the riskScore system.

### Validation of characteristic genes *in vivo* and *in vitro*

**Figure 11:**
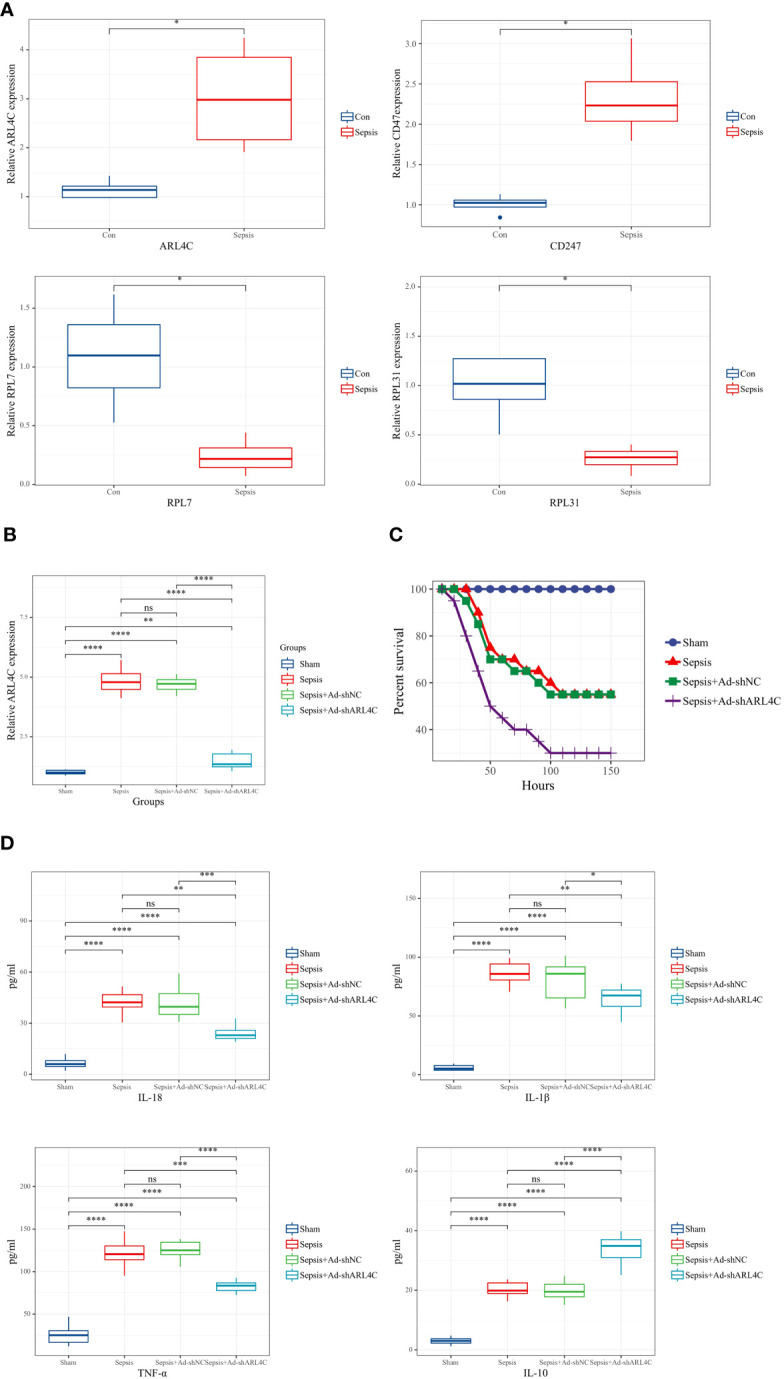
Validation of characteristic genes *in vivo* **(A)** Relative expression levels of DDR-related genes in control and sepsis groups (n=4 in each group). **(B)** Relative expression levels of ARL4C in control, sepsis, sepsis+Ad-shNC, and sepsis+Ad-shARL4C group (n=8 in each group). **(C)**Expression levels of inflammatory factors (IL-1β, TNF-α, IL-10, and IL-18) in the peripheral blood of rat with sepsis+Ad-shNC and sepsis+Ad-shARL4C (n=8 in each group). **(D)** Survival status of rats in each group (n=10 in each group). *p < 0.05, **p < 0.01, ***p < 0.001, ****p < 0.0001. NS, no significant difference.

To further corroborate the role of the characteristic genes in the construction of the riskScore model during sepsis, we designed validation experiments at both *in vivo* and *in vitro* levels. First, we established a sepsis model in rats and analyzed gene expression in their peripheral blood using RT-qPCR. A significant subset of genes, specifically ARL4C, CD247, RPL7, and RPL31, showed differential expression patterns consistent with the results obtained from our dataset (**Figure 11A**). We selected ARL4C for further analysis based on its possession of the largest absolute coefficient value. To assess the effectiveness of gene silencing in this model, RT-qPCR was employed again. Notably, in the Ad-shARL4C group, ARL4C expression decreased to approximately one-third of that observed in the sepsis+Ad-shNC group, validating the efficiency of gene silencing in our model (**Figure 11B**). In parallel, pro-inflammatory markers such as IL-1β, IL-18, and TNF-α were found to be upregulated in the Ad-shARL4C group, while the anti-inflammatory cytokine IL-10 was diminished (**Figure 11C**). These results point to ARL4C as a potential critical regulator in the immune and inflammatory responses during sepsis. Moreover, survival analysis indicated that rats with reduced ARL4C expression had lower survival rates in comparison to those in both the sham and sepsis+Ad-shNC groups (**Figure 11D**).

**Figure 12:**
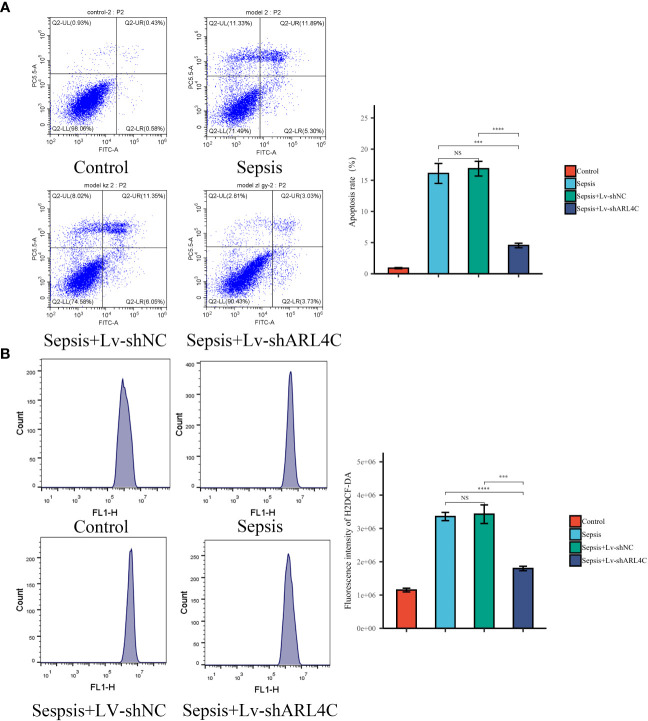
Validation of ARL4C *in vitro*. **(A)** Flow cytometry detected the apoptosis rate in the Control, Sepsis, Sepsis+Lv-shNC, and Sepsis+Lv-shARL4C groups. (n=4 in each group) **(B)** Flow cytometry detected the ROS production in the Control, Sepsis, Sepsis+Lv-shNC, and Sepsis+Lv-shARL4C groups. (n=4 in each group), ***p < 0.001, ****p < 0.001. NS, no significant difference.

To evaluate the impact of ARL4C inhibition on sepsis *in vitro*, lentivirus-shARL4C was introduced into RAW264.7 cells, and flow cytometry was employed to assess apoptotic levels and ROS production among the various groups. The ARL4C knockdown group showed increased LPS-induced apoptosis and ROS production rates (**Figures 12A, B**), suggesting that ARL4C blocking may make macrophages more susceptible to apoptosis and enhance the excessive inflammatory response during sepsis.

In this section, we further substantiate the role of characteristic genes, particularly ARL4C, in sepsis and demonstrate its protective function by attenuating excessive inflammation.

## Discussion

Sepsis characterized as a life-threatening organ dysfunction caused by a dysregulated host response to infection, constitutes a major global public health challenge. Despite recent advancements in treatment modalities, sepsis persists in carrying high rates of morbidity and mortality. The limitations in both research and practice hinder the potential for personalized treatments for sepsis patients. Therefore, a comprehensive understanding of the heterogeneity among sepsis patients and the disease’s diverse stages is pivotal in selecting suitable treatments and predicting accurate prognosis. Contemporary research suggests that a suitable DNA damage response (DDR) could effectively ameliorate sepsis by decreasing the pathogen burden, curbing excessive inflammatory reactions, and adeptly controlling immune responses (25–27). Despite the promising potential of DDR as a therapeutic target and prognostic predictor in sepsis, further exploration into the explicit molecular mechanisms and the associated genes implicated in these physiological and pathological processes is necessary.

In this study, we first employed scRNA-seq and Bulk RNA-seq analysis to systematically delve into the landscape of DDR in sepsis. By capitalizing on the synergistic advantages of both Bulk RNA-seq and scRNA-seq, we aim to devise an enhanced DDR-based risk score system for sepsis that promises improved diagnostic and therapeutic strategies for this critical condition. Intriguingly, we observed the enrichment of diverse forms of immune cells in patients at different disease stages and prognostic outcomes, suggesting that the recruitment and activation of various immune cell types can greatly influence the progression and prognosis of sepsis. Recent research further affirms the potential of peripheral blood immune cells as a distinctive marker of bacterial sepsis (28). Monocytes, megakaryocytes, CD4+ T cells, and neutrophils, compared to other immune cell types, exhibited higher DDR scores, thus underscoring their elemental role in DDR progression, aligning with findings from a 31 CRISPR-Cas9 screen-based genetic map of the human cell (29). Consistently, our transcriptomic and single-cell analyses demonstrated a reduced DNA damage response (DDR) score in sepsis patients when compared to control subjects. Furthermore, we observed a correlation between diminishing DDR scores and a decreased number of active immune cells concomitant with poorer disease prognosis, substantiating the protective role of DDR. In a novel finding, we identified a positive correlation between the decline in DDR scores and the recruitment and activation of various immune cell types, such as mast cells, natural killer (NK) cells, CD8+ T cells, B cells, and dendritic cells (DCs). Additionally, our analysis of intercellular communication revealed that monocytes and CD4+ T cells were crucial in the development of sepsis, notably in cases with elevated DDR levels. We also discerned an upregulation of the LGALS9-CD44/CD45 and RETN-CAP1 interactions in monocytes among the majority of samples with high DDR scores, whereas in samples with low DDR scores, an increased prevalence of MIF-CD74+CXCR4 and MIF-CD74+CD44 interactions was noted. Additionally, signaling pathways such as GALECTIN, BAFF, ANNEXIN, GRN, CSF, and IL-1 exhibited heightened activity in the high-DDR group. In conclusion, our study profoundly elucidates the complex interconnections within immune cells as a pivotal factor in the progression of DDR in sepsis, with monocytes posited to play an integral role in this progression. This groundbreaking discovery requires further in-depth mechanistic research.

Moreover, we identified 71 DDR-related genes by intersecting the feature genes derived from single-cell analysis and WGCNA. Subsequently, through LASSO Cox regression, we pinpointed four DDR-associated characteristic genes (ARL4C, CD247, RPL7, and RPL31). Notably, ARL4C, a member of the ADP-ribosylation factor family of GTP-binding proteins, plays a critical role in the infiltration of immune cells into the tumor microenvironment, as well as in various aspects of cancer invasion and proliferation (30–32). CD247 is a key gene impacting the prognosis of mouse sepsis (33) and has also been identified as a critical gene for septic shock (34). The ribosomal protein L7 (RPL7), recognized as a housekeeping gene, augments the nucleic acid chaperoning function of the HIV-1 Gag polyprotein. This enhancement effectively surmounts significant impediments inherent to the assembly of the virus (35). RPL31 is implicated in modulating the tumor immune microenvironment and has been recognized as a protective factor in breast cancer pathology (36). In our research, we innovatively designated these eight genes as characteristic genes of DDR in sepsis and constructed a DDR-riskScore system for the prognostic assessment of sepsis. We established ROC curves, survival curves, nomograms, calibration curves, and DCAs based on this scoring system, demonstrating it to be a reliable prognostic tool for sepsis.

Therefore, patients with sepsis were stratified into low- and high-risk categories according to the formulated riskScore system. Following the analysis of enriched biological functions and pathways in the two clusters, we characterized the low-risk group as bearing an “immune phenotype” due to relatively high DDR scores. In low-risk patients, the immune system is robust enough to protect against pathogens and prevent the worsening of infections. Conversely, high-risk individuals or those with a poor prognosis often experience immunosuppression at the onset of sepsis. Although the exact mechanisms underlying this immunosuppression are not fully understood, it is acknowledged as a significant factor in sepsis-related deaths due to the disruption of immune homeostasis. Sepsis-induced immunosuppression affects various cell types and functions, resulting in increased apoptosis in immune cells, T cell depletion, cellular changes through epigenetic remodeling, and reduced expression of surface molecules vital for activation (28, 37). These immune suppressive alterations have been implicated in a heightened susceptibility to secondary infections from opportunistic pathogens and viral reactivations, leading to detriments in prognosis and potentially death (38). A hallmark of immune suppression is the reprogramming of monocytes and macrophages, characterized by a diminished capacity to produce proinflammatory cytokines upon ex vivo stimulation with bacterial agonists, a phenomenon referred to as “LPS tolerance.” (39). Recent research has drawn attention to the close interaction between DDR and the immune system (40, 41). Our single-cell level analysis of sepsis samples found monocytes having high DDR scores and heightened intercommunication activities with immune cells, unsurprisingly noting that a high DDR score in immune cells is more prevalent in patients with favorable prognoses. Our immune infiltration analysis further demonstrated significantly higher immunological scores, immune cell infiltration, and immune modulators in the low-risk group. Thus, the low-risk group was identified as having an immune phenotype, while the high-risk group displayed an immune suppression phenotype.

In addition, we fashioned *in vivo* and *in vitro* sepsis models to validate the DDR-associated characteristic genes. We discovered that both CD247 and ARL4C exhibited high levels of expression in the bloodstream of the sepsis-afflicted rat, potentially serving as beneficial immune-inflammatory regulators. To corroborate this notion, we created a knock-down model and discerned that an enhanced immune-inflammatory response corresponded with a decrease in the expression levels of ARL4C in sepsis. Sepsis rats with ARL4C knock-down also exhibited a higher mortality rate. Various studies suggest that ARL4C plays a crucial role in intensifying tumor proliferation, invasion, and drug resistance via immune-related pathways, which include Wnt/β-catenin, AKT/mTOR, and MEK/ERK (42–44). Furthermore, recent studies have demonstrated the significant role of ARL4C in mediating immune-inflammatory responses within the tumor microenvironment. Elevated expression of ARL4C in immune cells, which is correlated with the activation of the cancer immune response, has been associated with unfavorable prognoses in several cancers, including bladder urothelial carcinoma, colon adenocarcinoma, kidney renal papillary cell carcinoma, lower-grade glioma, and uterine corpus endometrial carcinoma (45). The expression of ARL4C in cancer-associated stromal cells was notably higher, indicating that ARL4C may exert multiple effects on the tumor microenvironment (46). Moreover, increased expression of Arl4c has been associated with the activation of pancreatic stellate cells (PSCs) and increased drug resistance in pancreatic cancer. The induction of autophagy, mediated by the Yap-CTGF signaling pathway, is essential for Arl4c-related PSC activation (47). Currently, the majority of research on ARL4C is centered on the tumor immune microenvironment. However, the investigation of ARL4C’s role in sepsis remains notably scarce. In this study, we discovered that the knockdown of ARL4C in an *in vitro* sepsis model potentially renders macrophages more vulnerable to apoptosis and exacerbates the inflammatory response during sepsis. Overall, our study is the first to identify ARL4C’s involvement in the sepsis inflammatory response by affecting DDR, suggesting its significance in maintaining an appropriate inflammatory response.

This study has certain limitations, primarily attributable to its retrospective design and the relatively small sample size obtained from public databases, which necessitates additional validation of the results through multicentric prospective studies. Also, the stability of the results might be compromised as the transcriptomic data utilized were sourced from microarray datasets, potentially rendering them less consistent compared to those from *in vivo* or *in vitro* experiments. Moreover, to enhance the accuracy in determining the clinical utility for sepsis patients with diverse molecular subtypes and risk scores, it is advisable to utilize larger sample sizes. This approach would facilitate a more nuanced analysis of prognostic and therapeutic information.

## Conclusion

In our research, we executed an exhaustive evaluation of DDR expression patterns in sepsis, employing both single-cell and bulk transcriptomic analyses. Our findings indicate a potent connection between DDR activity levels and sepsis prognosis. Namely, patients exhibiting high DDR levels, enriched immune cells, and active immune responses displayed higher survival rates, in contrast to patients with lower DDR levels and immune suppression, who experienced higher mortality rates. Furthermore, we discerned four characteristic genes related to DDR and formulated a diagnostic and risk prediction model pertinent to sepsis. Such findings shed light on the DDR-related heterogeneity in sepsis, holding significant implications for the advent of personalized treatments and prognostic prediction in patients afflicted with this condition.

## Data availability statement

The original contributions presented in the study are included in the article/**Supplementary Material**. Further inquiries can be directed to the corresponding author.

## Ethics statement

The studies involving humans were approved by the Ethics Committee of the Second Affiliated Hospital of Fujian Medical University. The studies were conducted in accordance with the local legislation and institutional requirements. Written informed consent for participation was not required from the participants or the participants’ legal guardians/next of kin in accordance with the national legislation and institutional requirements. The animal study was approved by The Institutional Animal Care and Use Committee of The Second Affiliated Hospital of Fujian Medical University. The study was conducted in accordance with the local legislation and institutional requirements.

## Author contributions

GW: Conceptualization, Data curation, Formal analysis, Investigation, Project administration, Software, Visualization, Writing – original draft, Writing – review & editing. QL: Formal analysis, Validation, Writing – review & editing. RZ: Conceptualization, Investigation, Validation, Writing – review & editing. JY: Software, Visualization, Writing – review & editing. ZX: Writing – review & editing. SJ: Writing – review & editing.
