# Supplementary material for: Prognostic stratification of sepsis through DNA damage response based RiskScore system: insights from single-cell RNA-sequencing and transcriptomic profiling
Source: Front Immunol. 2024 Feb 9;15:1345321. doi: 10.3389/fimmu.2024.1345321 (PMC10884272; doi:10.3389/fimmu.2024.1345321)
Supplement: Supplementary file 1 [file DataSheet_1.docx]

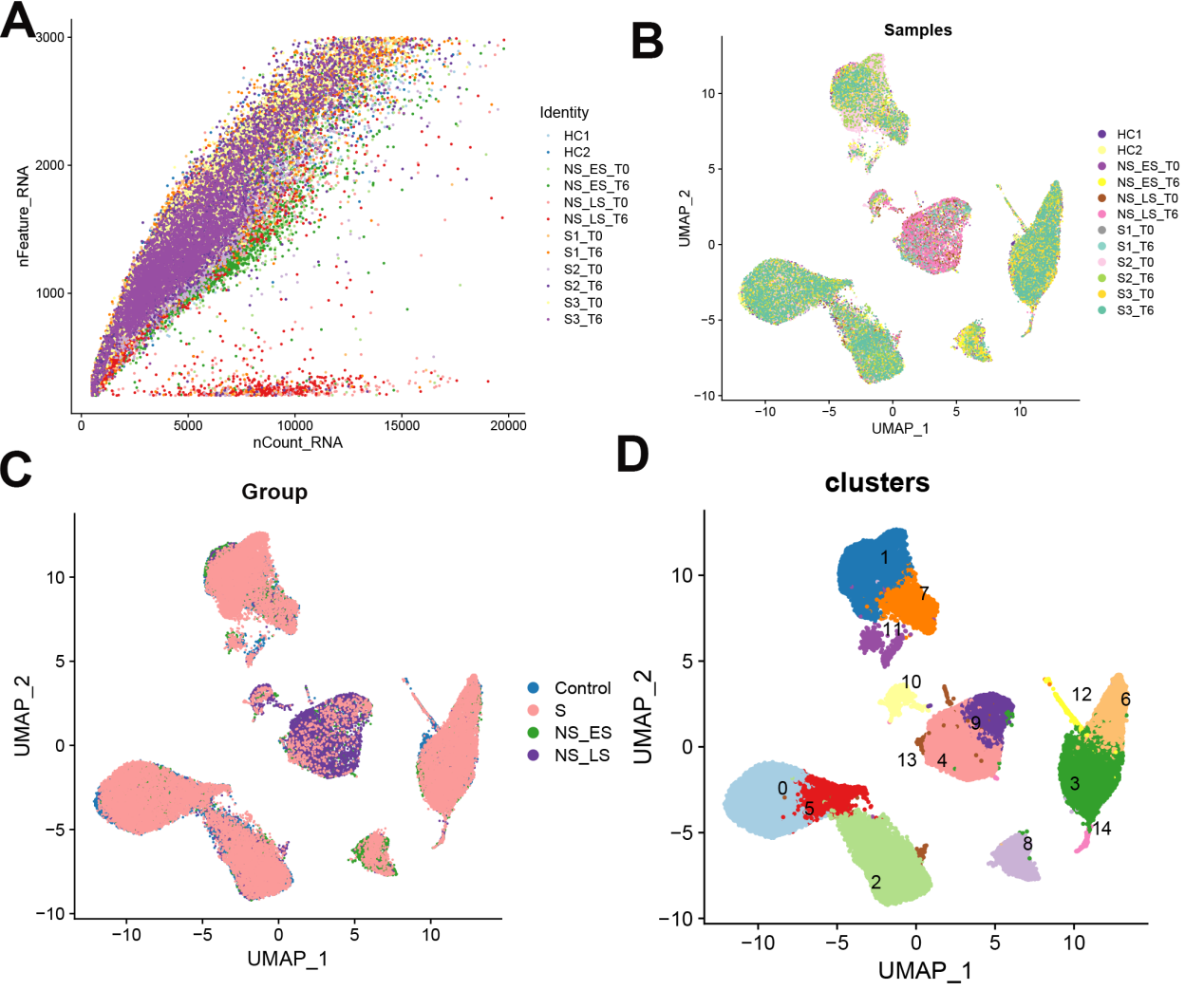


**Figure S1** **Annotation of single-cell data in GSE167363**. (A) Scatter plots revealing nFeature_RNA and nCount_RNA expressed per cell in all samples. (B) The UMAP projection of each group of cells. (C) Unified UMAP representation of all merged samples, with individual cells color-coded according to their respective conditions. (D) The UMAP projection of cell clusters.Each number represents a distinct cell subtype: "0"="CD8+ T cell", "1"="B cell", "2"="NK cell", "3"="Monocyte", "4"="Megakaryocyte", "5"="CD8+ T cell", "6"="CD4+ T cell", "7"="B cell", "8"="Neutrophil", "9"="Megakaryocyte", "10"="Mast cell", "11"="B cell","12"="DC", "13"="Megakaryocyte", and "14"="Monocyte".


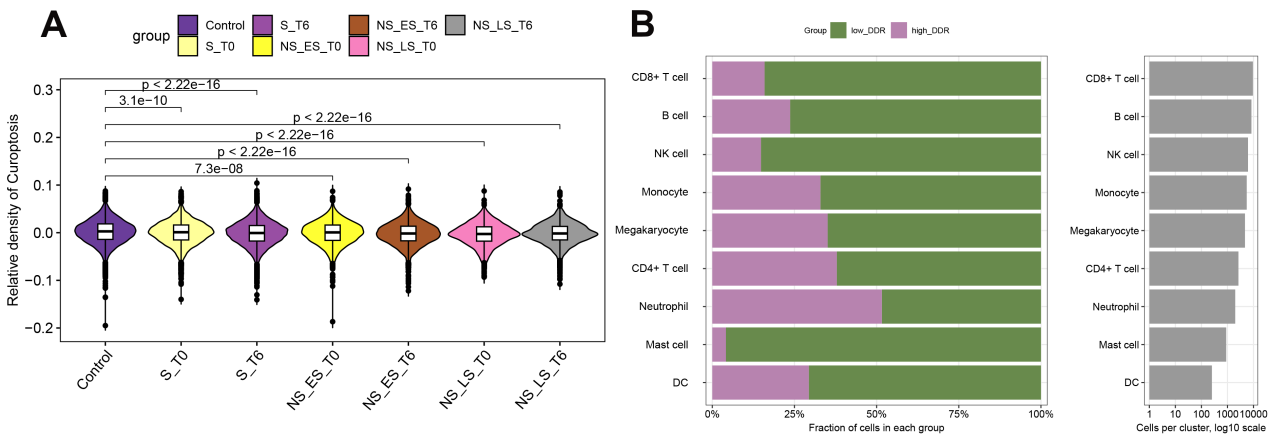


**Figure S2** (A) relative density of DDR in each group. (B) Cell type fractions of each group.


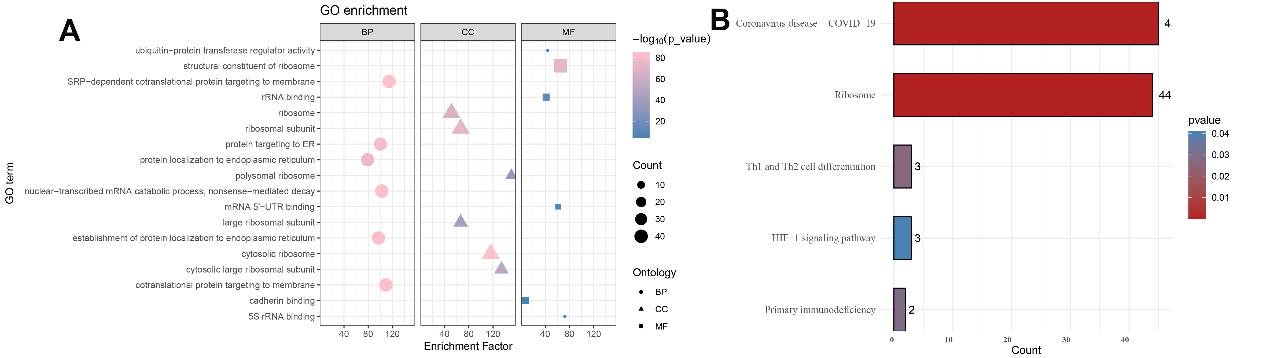


**Figure S3 [Enrichment analysis](javascript:;) of DDR-relate [characteristic](javascript:;) genes.** (A) GO analysis of 71 DDR-relate [characteristic](javascript:;) genes. (B) KEGG analysis of 71 DDR-relate [characteristic](javascript:;) genes.


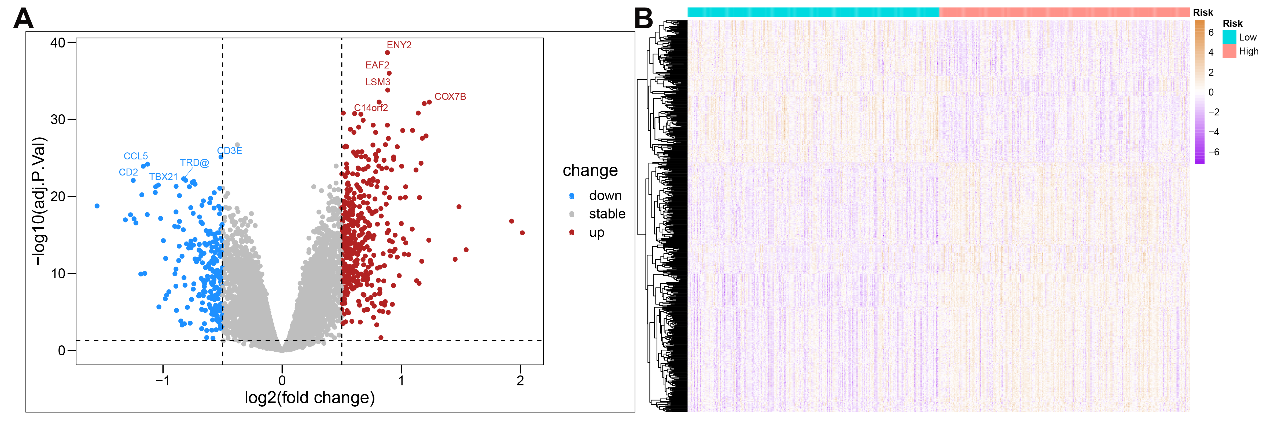


**Figure S4 feature genes of high- and low-risk group**.


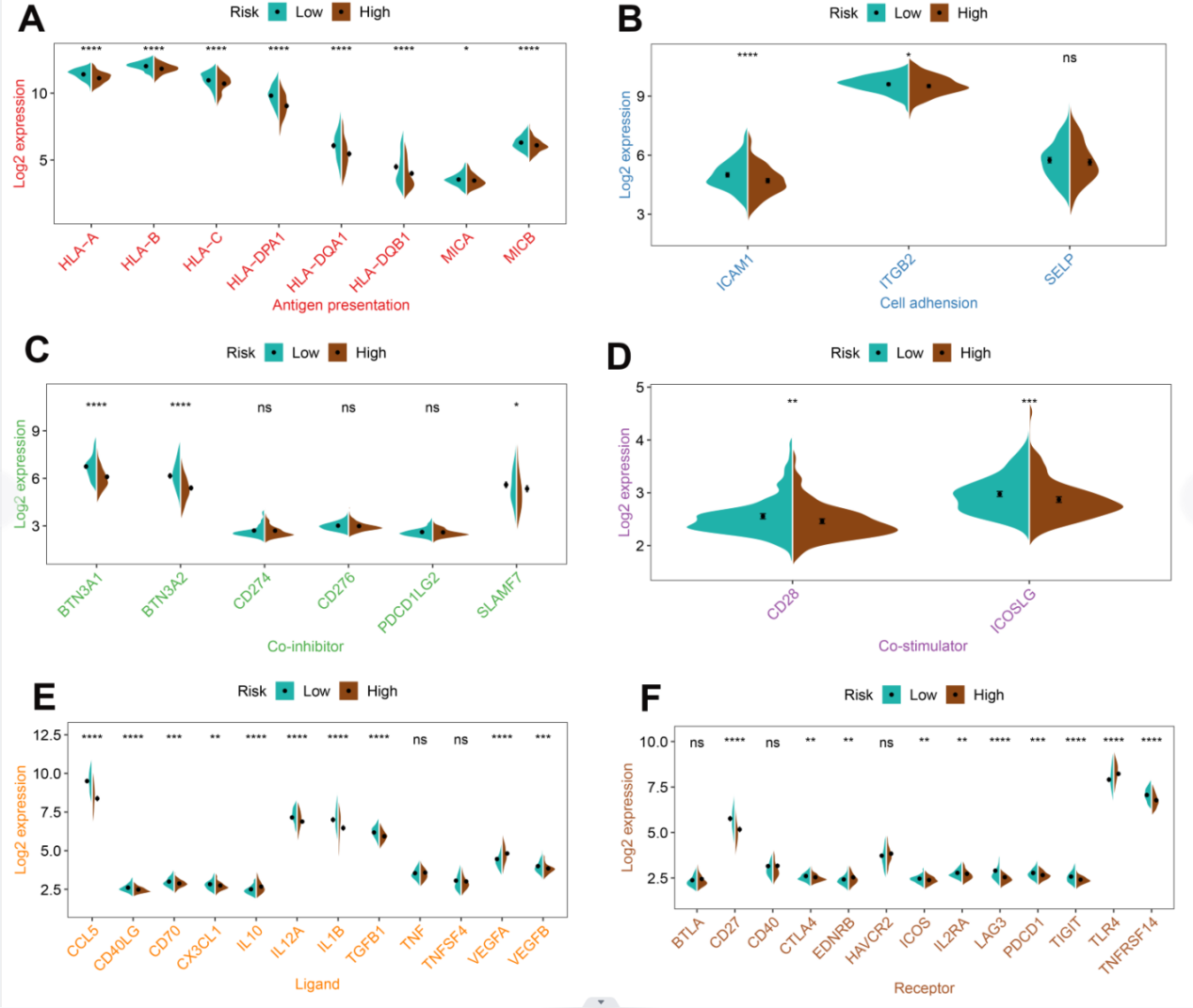


**Figure S5 the expression profiles of immunoregulatory subgroup genes in sepsis patients at low and high risk.** *p < 0.05, **p < 0.01, ***p < 0.001, ****p < 0.0001.
